# Dataset of two-phase flow in a horizontal pipe: synchronized measurements of acceleration, pressure, void fraction and high-velocity camera

**DOI:** 10.1016/j.dib.2025.112117

**Published:** 2025-10-03

**Authors:** Daniely A. das Neves, Saon C. Vieira, Juliana R. Cenzi, Adriano T. Fabro, Bernardo P. Foresti, Marcelo S. Castro

**Affiliations:** a Center for Energy and Petroleum Studies, Universidade Estadual de Campinas, Campinas-SP 13083-970, Brazil; b Petrobras, Santos-SP, Brazil; c School of Mechanical Engineering, Universidade Estadual de Campinas, Campinas-SP 13083-860, Brazil; d Department of Mechanical Engineering, University of Brasília, Brasília-DF 70910-900, Brazil

**Keywords:** Two-phase flow, Horizontal pipes, Void fraction, Structural vibration, High-velocity camera

## Abstract

This dataset provides a valuable resource for the detailed study of two-phase flows and flow-induced vibrations in pipes, essential in sectors such as oil, chemical, and nuclear industries. Data collection and analysis were conducted at the Experimental Laboratory of Petroleum (LabPetro) within the Center for Energy and Petroleum Studies (CEPETRO) at the *Universidade Estadual de Campinas* (UNICAMP). The experimental data comprises 23 points for a gas-water mixture flowing in a horizontal pipe, representing three flow patterns: stratified, slug, and dispersed bubble. To capture the complex characteristics of the flow, synchronized data from void fraction sensors, piezoelectric pressure sensors, and accelerometers were acquired for 600 seconds at a sampling rate of 25.6 kHz. In addition, high-velocity videos were recorded at 800 fps for approximately 15 seconds, also synchronized with the sensors data. A deep learning algorithm was applied to segment the bubbles in the images, allowing accurate identification of their contours. Image processing provided information on the contours, diameters, number, and mean diameter of the smaller bubbles in all recorded images. Additionally, for some frames, a manual image segmentation process was performed to obtain accurate information on the location and contour of each bubble, enabling tracking and calculation of the individual velocity of each bubble. The integration of sensor data with image processing results and bubble tracking allows for a comprehensive analysis of the multiphase flow dynamics. In conclusion, two databases were produced from this experimental data. The first, $Dataset_{25.6kHz}$, contains the raw time series from the sensors (pressure, acceleration, void fraction) and the trigger signal, all at a sampling rate of 25.6 kHz. The second, $Dataset_{800Hz}$, was generated by downsampling the first to 800 Hz to match the camera's frame rate (800 fps). This final database integrates the sensors data with the image processing results, including the corresponding frame, number of bubbles, diameters, contours, segmentation, and bubble tracking information.

Specifications Table

**Table:** 

Subject	Engineering
Specific subject area	Two-phase gas-water flow in horizontal pipe
Type of data	Data in pickle format (.pkl) and images in tagged image file format (.tif)
Data collection	Data acquisition was conducted in a horizontal steel pipe conveying an air-water mixture, using tap water and compressed air as working fluids. The instrumentation included 10 piezoelectric pressure sensors (PCB 112B21 and PCB 106B50 models) with flush and pinhole mounts, 5 accelerometers (PCB 352C33 model), and a set of conductance-based void fraction meters with 4 measurement stations. The signals from these sensors were acquired synchronously at 25.6 kHz for 600 s using a National Instruments (NI) system with a LabVIEW interface. Simultaneously, a high-velocity camera (Phantom VEO 640 model) recorded images at 800 fps for approximately 15 s. To enable direct synchronization between the sensor data and the images, the 25.6 kHz dataset was downsampled to generate a secondary 800 Hz dataset. The final synchronization was ensured by a trigger signal.
Data source location	Experimental Laboratory of Petroleum – Artificial Lift and Flow Assurance Research Group, Center for Energy and Petroleum Studies, Universidade Estadual de Campinas, Barão Geraldo/Campinas-São Paulo- Brazil
Data accessibility	Repository name: Dataset of two-phase flow in a horizontal pipe: synchronized measurements of acceleration, pressure, void fraction and high-velocity camera - Raw Data Direct URL (Dataverse): https://doi.org/10.25824/redu/ISMWP4 Direct URL (Unidrive): https://unidrive.unicamp.br/index.php/s/LSGjo8bnfnT6cEX Access information: The dataset is centrally stored within the university's research data infrastructure. Access is provided through the Dataverse URL, which serves as the main interface, offering metadata and links to the complete dataset. Due to the dataset's large file size, the data files are stored within the central infrastructure through the Unidrive tool, ensuring efficient management. Furthermore, given the volume of the data, the use of a download manager is recommended.
Related research article	Neves, D. A., Vieira, S. C., Cenzi, J. R., Paula, A. d., Castro, M. S. & Fabro, A. T. (2024). Identification of the flow pattern from the experimental pressure signal in horizontal pipes carrying two-phase flows. Experimental Thermal and Fluid Science. DOI:https://doi.org/10.1016/j.expthermflusci.2024.111141. Neves, D. A., Fabro, A. T., Vieira, S. C., Cenzi, J. R. & Castro, M. S. (2023). An experimental investigation on the fluid-structure coupling in horizontal pipes conveying two-phase intermittent flow. International Journal of Multiphase Flow. DOI: https://doi.org/10.1016/j.ijmultiphaseflow.2024.104825 Neves, D. A., Vieira, S. C., Cenzi, J. R., Barbosa, B. R., Castro, M. S. & Fabro, A. T. (2024). Development and Dynamic Characterisation of a Conductance-based Void Meter Array. Measurements. DOI: https://doi.org/10.1016/j.measurement.2024.115923.

## 1 Value of the Data

The dataset outlined in the introduction holds significant value for the scientific community, particularly in the fields of petroleum, chemical, and nuclear industries interested in two-phase, characterization of internal flow, Flow Induced Vibration (FIV), Fluid-Structure Interaction (FSI) and turbulence, and other related issues, in pipes at various frequencies. The dataset's importance is underscored by:

• **Comprehensive understanding of flow and vibration:** the dataset goes beyond providing temporal and spatial details of the flow through pressure sensors, acceleration sensors, void fraction measurements, and flow recordings. It also captures induced vibration in the pipeline. This comprehensive perspective is essential for a profound understanding of the intricate dynamics of two-phase flow.
• **Spectral model development:** the dataset serves as a valuable resource for further exploration of spectral models related to pipe excitation caused by two-phase flow. Researchers can use these data to refine and develop models that elucidate the complex dynamics of pipe vibrations.
• **Characterization of internal flow:** the dataset includes measurements of fluctuation pressure, void fraction, and recordings of internal flow, enabling the detailed characterization of internal flows. This facet is essential for researchers aiming to comprehend the correlation between flow patterns and the fundamental dynamics of two-phase flow within pipelines.
• **Studies of fluid/structure interaction:** the dataset, encompassing measurements across a broad frequency range that includes structural vibration signals, dynamic pressure, and void fraction, across four prevalent flow pattern conditions, serves as a valuable asset for in-depth investigations into fluid/structure interaction within horizontal tubes. The dataset enables a comprehensive analysis of the system's inputs and outputs through these sensors. Researchers can explore the intricacies of this interaction, providing valuable insights into engineering applications and potential avenues for optimization.
• **Detailed analysis of bubble behavior:** the synchronization of high-sampling-rate images with other sensors in the flow enables a meticulous and accurate analysis of bubble behavior in response to flow fluctuations, as well as the distribution of bubble diameters. This integration facilitates refinement in model formulation, representing a significant tool for assessing the reliability of measurements.

## 2 Background

Studies involving two-phase flow are of paramount importance to the oil industry due to its presence in various processes across the entire production system, from reservoirs to refining [[1], [2], [3], [4]]. Therefore, understanding the phase configuration throughout the flow is crucial, as the distribution of phases dictates the behavior of pressure gradients and quantities such as heat transfer coefficients in the mixture. Moreover, the oscillatory behavior of multiphase flow can lead to structural vibrations and potentially result in line ruptures and structural problems [[5], [6], [7]]. Hence, identifying and characterizing multiphase flows is of extreme importance.

This comprehensive dataset establishes a critical foundation for in-depth investigations into flow characterization, Flow-Induced Vibration (FIV), Fluid-Structure Interaction (FSI), and flow pattern identification. It is especially well-suited for the development and validation of data-driven models, ranging from classic machine learning to deep neural networks. Specifically, for image analysis, the provided data is not limited to the application of the Segment Anything Model (SAM) [8], but also encourages the exploration of novel processing methodologies, offering a solid basis for benchmarking and performance comparison.

Among the main sensors used in experimental studies of two-phase flow, the literature highlights the use of pressure sensors and structural vibration sensors [6,9]. In the case of pressure sensors, they provide direct and accurate measurements, providing valuable information about dynamic pressure for different flow patterns. The use of structural vibration sensors, in addition to being non-invasive, stands out for enabling the monitoring of the system's dynamic behavior without the need for direct insertion into the flow [5,10,11]. This non-intrusive characteristic is particularly relevant in situations where interference in the flow must be minimized, ensuring more representative measurements and preserving the natural conditions of the flow.

The accurate monitoring of two-phase flows in controlled environments is essential for understanding underlying physical phenomena, validating existing models, and developing new measurement instruments. Intrusive sensors, such as resistive (or conductance) sensors and Wire Mesh Sensors (WMS), play a fundamental role in laboratories, investigating critical variables such as void fraction and bubble size [12,13]. This dataset provides measurements of void fraction obtained by resistive sensors for different flow patterns. Additionally, a new sensor has been developed in the laboratory with an innovative configuration of resistive sensors, addressing crosstalk issues, employing a demodulation algorithm and robust calibration, as detailed in Das Neves [12] work. Validated in experimental environments, the sensor array demonstrates linearity and gain, being crucial for the dynamic characterization of void fraction. In industrial contexts, precise monitoring of two-phase flows is vital, emphasizing the ongoing importance of intrusive sensors in the validation and improvement of measurement techniques.

The objective of this database is to facilitate the investigation of the dynamics of two-phase flow in horizontal pipelines. To achieve this, synchronized data from enhanced, cross-talk-free void fraction sensors, piezoelectric pressure sensors, and structural vibration sensors are provided. The dataset also includes high-sampling-rate images from flow recordings, offering synchronization capabilities with sensor data. Furthermore, the incorporation of image processing data, utilizing the SAM methodology, provides valuable insights into bubble diameter, the number of bubbles per image, and their respective contours. This comprehensive dataset aims to contribute significantly to advancements in understanding and optimizing multiphase flows in industrial applications.

## 3 Data Description

The datasets are built upon 23 experimental points, generated by the systematic variation of the superficial velocities of the liquid ($j_{sl}$) and gas ($j_{sg}$) phases. As detailed in Table 1, the liquid superficial velocity ranges from 0.258 to 3.328 m/s, while the gas superficial velocity ranges from 0.078 to 2.036 m/s. This matrix of conditions resulted in the formation of two primary two-phase flow patterns: Slug (SL) and Dispersed Bubble (DB). The classification of these patterns was performed using a dual-method approach, based on both high-velocity camera images and data from the void fraction sensor, as explained in detail in Section 4.1. Throughout all experiments, the fluid properties (water and air) and the physical configuration of the pipeline were held constant.

**Table 1:** Experimental test matrix with liquid superficial velocities ($j_{sl}$), gas superficial velocities ($j_{sg}$), and flow pattern classification using high-velocity camera and void fraction sensor. The flow patterns are classified as Slug (SL), and Dispersed Bubble.

Point	Flow pattern (image)	Pattern (void fraction sensor)	$J_{sl}$ [m/s]	$J_{sg}$ [m/s]
1	–	SL	0.258	0.106
2	–	SL	0.259	0.203
3	–	SL	0.258	0.481
4	SL	SL	0.258	0.899
5	–	SL	0.258	1.146
6	SL	SL	0.258	1.821
7	–	SL	0.259	2.036
8	SL	SL	0.580	0.504
9	SL	SL	0.579	0.977
10	SL	SL	0.578	1.429
11	SL	SL	1.191	0.382
12	SL	SL	1.315	0.845
13	SL	SL	1.353	1.399
14	SL	SL	1.300	0.493
15	SL	SL	1.242	1.001
16	SL	SL	1.212	1.218
17	SL	SL	2.150	0.532
18	SL	SL	2.044	1.017
19	SL	SL	2.017	1.182
20	DB	DB	3.153	0.095
21	SL	SL	3.042	0.421
22	SL	SL	2.927	0.854
23	DB	DB	3.328	0.078

The experimental data is organized into two distinct, synchronized datasets. The first, $Dataset_{25.6kHz}$ containing time series data acquired at 25.6 kHz from 4 void fraction sensors, 5 accelerometers, 10 pressure sensors, and the high-velocity camera trigger signal. The second dataset, $Dataset_{800Hz}$, contains the sensor time series from the first set, which were downsampled to 800 Hz for alignment with the camera's acquisition rate. Crucially, this dataset is augmented with extensive image processing results, such as time series of the number of bubbles, average and equivalent diameters, and bubble contour data for each frame, in addition to data originating from manual bubble segmentation and tracking.

The variables present in $Dataset_{25.6kHz}$, in the files that contain the data with time series from the sensors are:

□ *Time [s] (float64):* represents the time series associated with the acquisition of experimental data. Each entry corresponds to the time elapsed since the start of the experiment, in seconds.
□ *R1 [-]* ***-*** *R4 [-] (float64):* void fraction signals measured at different points along the experimental bench.
□ *Ac1 [m/s²]* **-** *Ac5 [m/s²] (float64)*: acceleration signals measured at different points along the experimental bench.
□ *Pre1 [Pa] - Pre10 [Pa] (float64):* pressure signals measured at different positions along the experimental bench.s
□ *Trigger [V] (float64):* trigger signal used for synchronizing the sensor signals with the high-velocity camera.

The $Dataset_{800Hz}$ includes all variables from the $Dataset_{25.6kHz}$, downsampled to 800 Hz, and augmented with the following image processing-derived data:

□ *Frame [-] (str):* text identifier associated with each frame captured during the experiment. This variable is used to track and correlate information from processed images.
□ *Number of bubbles [-] (float64):* number of bubbles detected in each frame during the image processing.
□ *Average diameter [mm] (float64):* average diameter of the bubbles detected in each frame, calculated from the image processing.
□ *Equivalent diameter [mm] (ndarray):* equivalent diameter of individual bubbles, stored as an array. Each value in the array represents the diameter of a specific bubble.
□ *Bubble contours [-] (ndarray):* contours of each bubble detected in each frame. This variable is represented as a multidimensional array containing the coordinates of the vertices that define the contours.
□ *Segmentation of bubbles [-] (dictionary):* dictionary with manual bubble segmentation. This dictionary includes the following variables:
  ▪ *ID of segmented bubble [-] (ndarray):* a unique identifier assigned to each bubble. This ID is used to track and individually identify each bubble.
  ▪ *Angle of the segmented bubble [pixels] (ndarray):* the angular measurement associated with the orientation or direction of a bubble within a reference coordinate system.
  ▪ *CxCy of the segmented bubble [pixels, pixels] (ndarray):* centroids of the bubble, which correspond to the coordinates of the geometric center of the bubble.
  ▪ *DaDb of the segmented bubble [pixels, pixels] (ndarray):* principal axes of an ellipse fitted to the bubble shape. These values describe the major and minor axes of the best-fit ellipse, representing the bubble's geometric dimensions.
□ *Tracking of bubbles [-] (dictionary)*: dictionary with manual bubble tracking. This dictionary includes the following variables:
  ▪ *ID of the bubble in reference frame [-] (ndarray):* a unique identifier assigned to each bubble in the reference frame.
  ▪ *CxCy of the bubble in reference frame [pixels, pixels] (ndarray):* centroid coordinates of the bubble in the reference frame, which correspond to the coordinates of the geometric center of the bubble.
  ▪ *DaDb of the bubble in reference frame [pixels, pixels] (ndarray):* principal axes of an ellipse fitted to the bubble shape in the reference frame.
  ▪ *Angle of the bubble in reference frame [pixels] (ndarray)*: angular orientation of the bubble in the reference frame.
  ▪ *ID of the bubble in current frame [-] (ndarray):* a unique identifier assigned to each bubble in the current frame.
  ▪ *CxCy of the bubble in current frame [pixels, pixels] (ndarray):* centroid coordinates of the bubble in the current frame.
  ▪ *DaDb of the bubble in current frame [pixels, pixels] (ndarray):* principal axes of the ellipse fitted to the bubble in the current frame
  ▪ *Angle of the bubble in current frame [pixels] (ndarray):* angular orientation of the bubble in the current frame
  ▪ *ID of the tracked bubble [-] (ndarray):* a unique identifier for each bubble tracked between the current frame and the reference frame
  ▪ *CxCy of the tracked bubble [pixels, pixels] (ndarray):* centroid coordinates of the tracked bubble.
  ▪ *DaDb of the tracked bubble [pixels, pixels] (ndarray):* principal axes of the ellipse fitted to the tracked bubble.
  ▪ *Angle of the tracked bubble [pixels] (ndarray):* angular orientation of the tracked bubble
  ▪ *Velocity of the tracked bubble - magnitude [m/s] (ndarray):* magnitude of the velocity of the tracked bubble.
  ▪ *Velocity of the tracked bubble - vertical [m/s] (ndarray):* vertical component of the tracked bubble's velocity.
  ▪ *Velocity of the tracked bubble - horizontal [m/s] (ndarray):* horizontal component of the tracked bubble's velocity
  ▪ *Area of the tracked bubble [mm²] (ndarray):* surface area of the tracked bubble.

Both databases provide comprehensive metadata for each experimental point, as summarized in Table 2. This metadata encompasses fluid properties (densities, viscosities, and surface tension), flow characteristics (superficial velocities, temperature, pressures and void fraction with quick-closing valves technique), detailed about the pipe (internal diameter, thickness, inclination, density, elastic modulus and roughness) and the pixel/mm ratio for the high-velocity camera images is provided for accurate spatial measurements. To achieve both low and high flow velocities, two types of pumps were utilized: centrifugal (CC) and positive displacement (PD). The specific pump employed for each experimental point is indicated in the metadata.

**Table 2:** Variables present in the metadata.

Variable	Unit
Experimental point	–
Pattern (image)	–
Pattern (void fraction sensor)	–
Liquid superficial velocity ($J_{sl}$)	m/s
Gas superficial velocity ($J_{sg}$)	m/s
Pump	–
Mean temperature	°C
Mean absolute pressure	Pa
Pressure gradient	Pa/m
Void fraction in quick-closing (image)	–
Void fraction in quick-closing (void fraction sensor)	–
Void fraction in quick-closing (fluid drainage)	–
Density - liquid phase	kg/m³
Density - gas phase	kg/m³
Viscosity - liquid phase	Pa.s
Viscosity - gas phase	Pa.s
Superficial tension	N/m
Internal diameter - pipe	m
Thickness - pipe	m
Inclination - pipe	Rad
Density - pipe	kg/m³
Modulus of elasticity - pipe	GPa
Roughness - pipe	–
Scale - images	mm/pixel

### 3.1 Dataset_25.6 kHz_

**Fig. 1:**
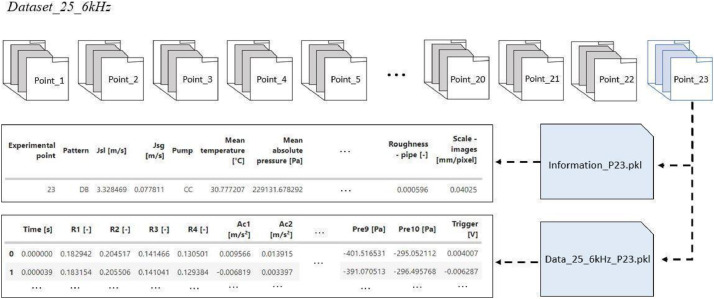
Illustration of the $Dataset_{25.6kHz}$ database configuration, where there are 23 experimental points distributed in a folder, in each folder there is a metadata file with information on fluid, flow and structure properties and a file containing the sensor time series.

The first database is organized into 23 folders, each corresponding to a distinct experimental point, as present in Fig. 1. Within each folder, two pickle-format files are stored: a metadata file and a data file. The metadata file contains comprehensive information on fluid properties, flow characteristics, and structural parameters as previously described. The data file holds time series for piezoelectric pressure ($P_{re1}$- $P_{re10}$), acceleration ($A_{c1}$- $A_{c2}$), void fraction ($R_{1}$-$\;R_{2}$), and the triggering of the high-velocity camera, all acquired at a 25.6 kHz sampling rate for 600 s. Details regarding the specifications, model, manufacturer, and positioning of each sensor are provided in Section 4.

Superficial velocities of the liquid and gas phases were determined via mass flow rate measurements, obtained using Coriolis flow meters, divided by the respective phase density and pipe cross-sectional area. Liquid density (water) was considered constant at 998 kg/m³. Gas phase density was calculated using the ideal gas law with mean pressure and temperature values. The mean temperature was determined from an average of sensors positioned at the inlet and outlet of the test section, while the mean pressure was derived from sensors located at the inlet, outlet, and within the test section, as shown in Fig. 8. Fluid viscosity and surface tension values were obtained from the literature [14]. Flow pattern identification was conducted through analysis of high-velocity camera images and void fraction sensor data, with detailed discussion provided in subsequent sections. Temperature measurements, averaged from two sensors positioned upstream and downstream of the test section (Fig. 8), were used in conjunction with absolute pressure measurements, averaged from sensors at the inlet, outlet, and test section, to determine gas phase density. For experimental points 8–19, 21, and 22, characterized by slug flow and high passage frequency, a differential pressure sensor across the test section provided pressure gradient measurements. Void fraction was determined using the quick-closing valves method, enabling rapid isolation of a pipe section and accurate capture of the instantaneous gas-liquid distribution. The trapped gas volume following valve closure was measured using high-velocity camera images, void fraction sensor data, and fluid drainage techniques, details will be discussed in subsequent sections.

**Fig. 2:**
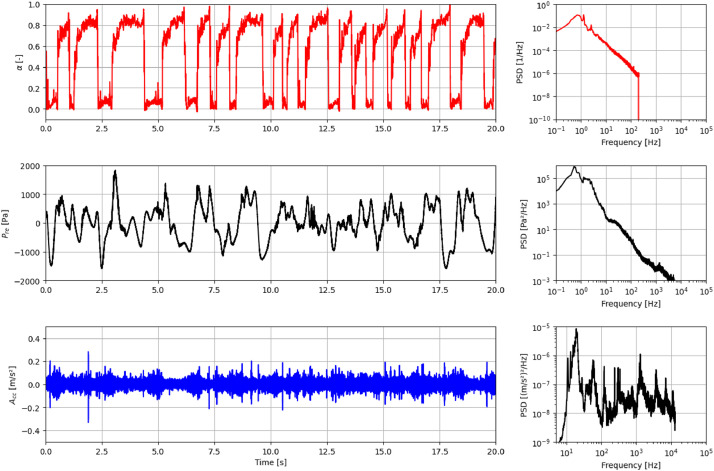
Time series and corresponding Power Spectral Density (PSD) data for experimental point 13, encompassing void fraction (α), pressure ($P_{re}$), and acceleration ($A_{cc}$).

The Nyquist theorem establishes that to avoid aliasing and accurately reconstruct the signal, the sampling rate must be at least twice the maximum frequency present in the signal [15,16]. Therefore, with a 25.6 kHz sampling rate, dynamic events and transient phenomena up to 10 kHz in frequency can be reliably analyzed. This high sampling rate is essential for capturing fast, high-frequency variations in the system, ensuring that no significant information is lost. For example, Fig. 2 presents the Power Spectral Density (PSD) of the void fraction, pressure and acceleration signals. High-frequency phenomena, consistent with the fluid-structure coupling analysis presented in Das Neves [1], are evident in the acceleration and pressure signals. Conversely, the void fraction signal exhibits a limited frequency range, which is a direct consequence of its acquisition method. Although the measurement system has a high overall sampling rate of 25.6 kHz, it utilizes a switching frequency of 400 Hz to alternate the reading among the four different sensors. This strategy results in an effective acquisition frequency of 400 Hz for each individual sensor. According to the Nyquist Theorem, this effective rate limits the maximum frequency of the phenomenon that can be accurately analyzed to 200 Hz, thus explaining the energy content observed in its PSD.

### 3.2 Dataset_800Hz_

**Fig. 3:**
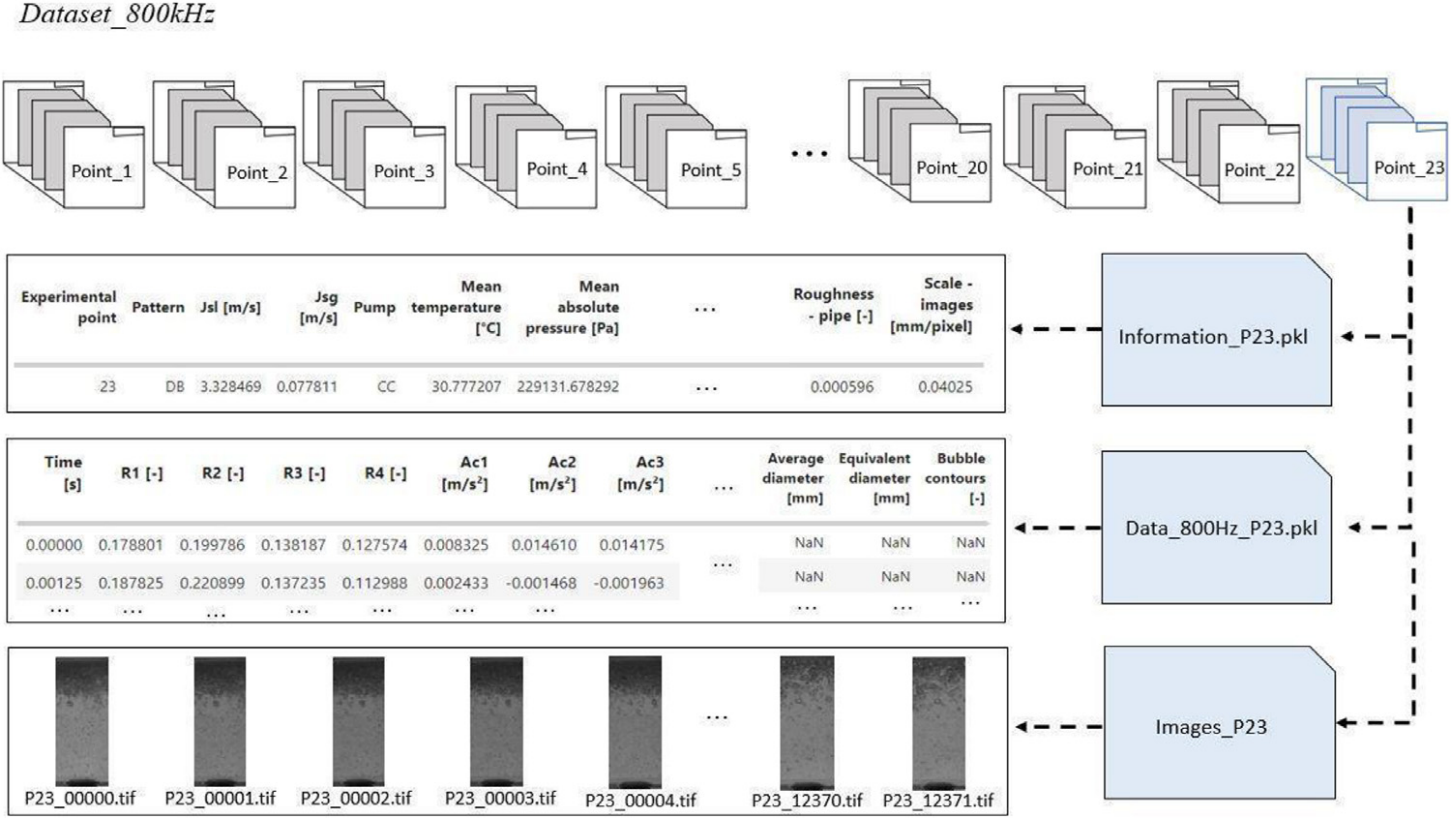
Illustration of the $Dataset_{800Hz}$ database configuration, where there are 23 experimental points distributed in a folder, in each folder there is a file information about the experimental point, a file that contains the time series of the sensors and results of image processing and folder with images of the flow.

In the second dataset, the sensor time series is downsampled to 800 Hz to match the sampling rate of the images provided by the high-velocity camera, which was set at 800 fps. Note in Fig. 3 that within each folder of an experimental point, there are three files: one containing information about the experimental point, the metadatas previously described, another containing the time series data from the sensors (10 pressure, 5 acceleration, and 4 void fraction sensors), and resulting image processing (frame, number of bubbles, average diameter, equivalent diameter, bubble contours, segmentation of bubbles, and tracking of bubbles), and the third is a folder with images of the flow, saved in TIFF format, totaling 12,372 frames per experimental point, corresponding to approximately 15 s of recorded images. The variable named ``number of bubbles'' and ``average diameter'' represents the total number of bubbles and the average bubble diameters present in each frame. Since ``equivalent diameter'' and ``bubble contours'' are lists that contain, respectively, the equivalent diameters and the coordinates of the contour of each bubble in each frame. Due to the high sampling rate in the flow recording, it was possible to record for a duration of 15 s. However, the time series data from the sensors have a duration of 600 s. Therefore, in periods where there is no image recording, Not a Number (NaN) values were added to the dataframe. It is important to note that the synchronization between the camera recording and the time series data from the other sensors was controlled using a trigger signal that indicates the point in the time series when the camera started recording the flow.

**Fig. 4:**
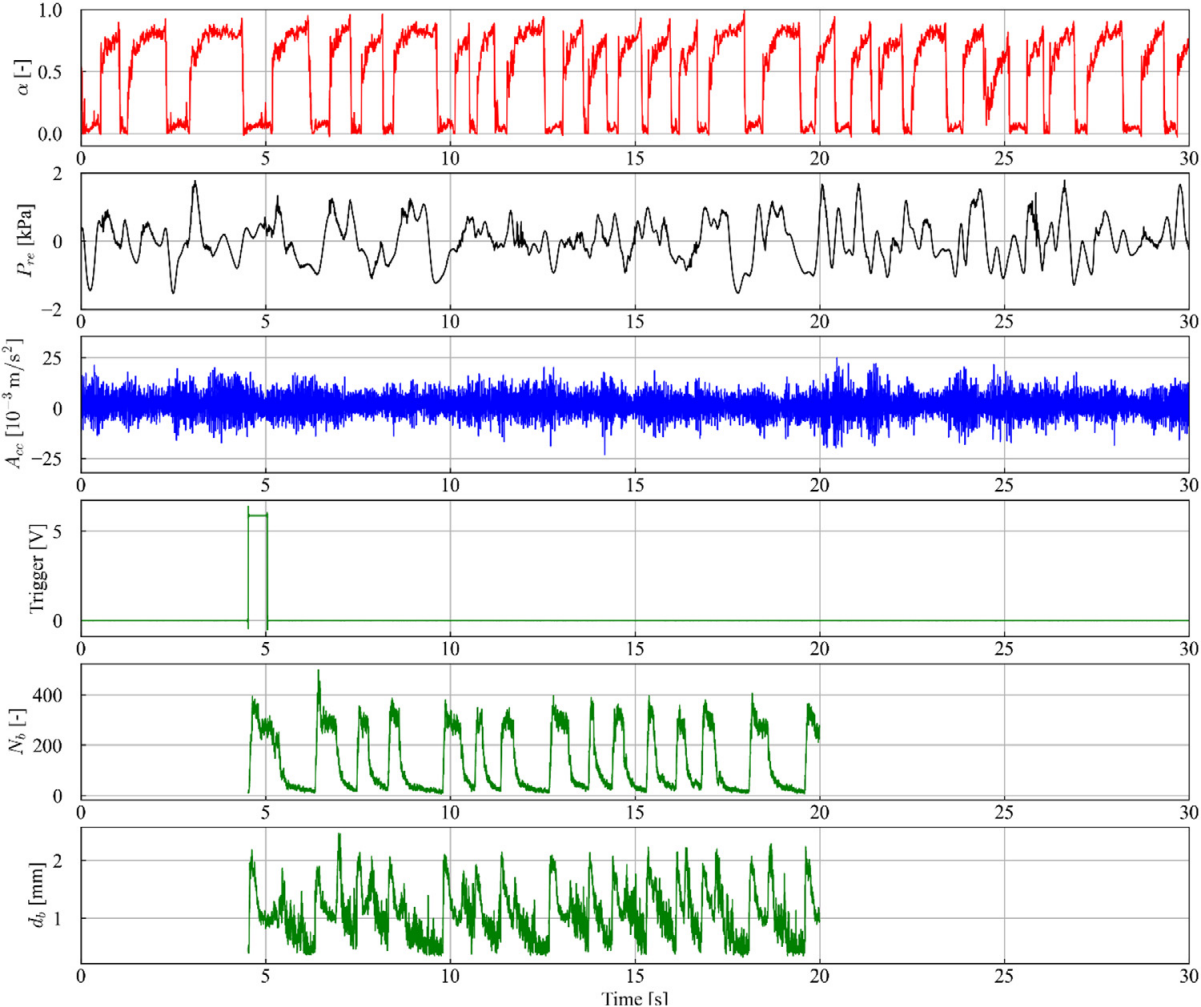
Time series data for experimental point 13, encompassing void fraction (α), pressure ($P_{re}$), acceleration ($A_{cc}$), number of bubbles ($N_{b}$), and average diameter ($d_{b}$).

In Fig. 4, 30 s of the temporal series from the sensors of void fraction, piezoelectric pressure, and acceleration sensor are presented, along with the trigger signal that initiates the recording of the high-velocity camera. The series of bubble count and average diameter obtained through image processing with the Segment Anything Model (SAM) is also presented, further details regarding the method are presented in Section 4.3. It is observed that the series of bubble count and average bubble diameters have a shorter duration than those of the sensors, due to the recording capacity of approximately 15 s with the high-velocity camera. Additionally, it is noteworthy that the recording starts with a high trigger value. As mentioned before, “NaN” values were added to the variables “Frame [-]”, “Number of bubbles [-]”, “Average diameter [mm]”, “Equivalent diameter [mm]”, “Bubble contours [-]”, “Segmentation of bubbles [-]” and, “Tracking of bubbles [-]” in intervals where there is no recording.

**Fig. 5:**
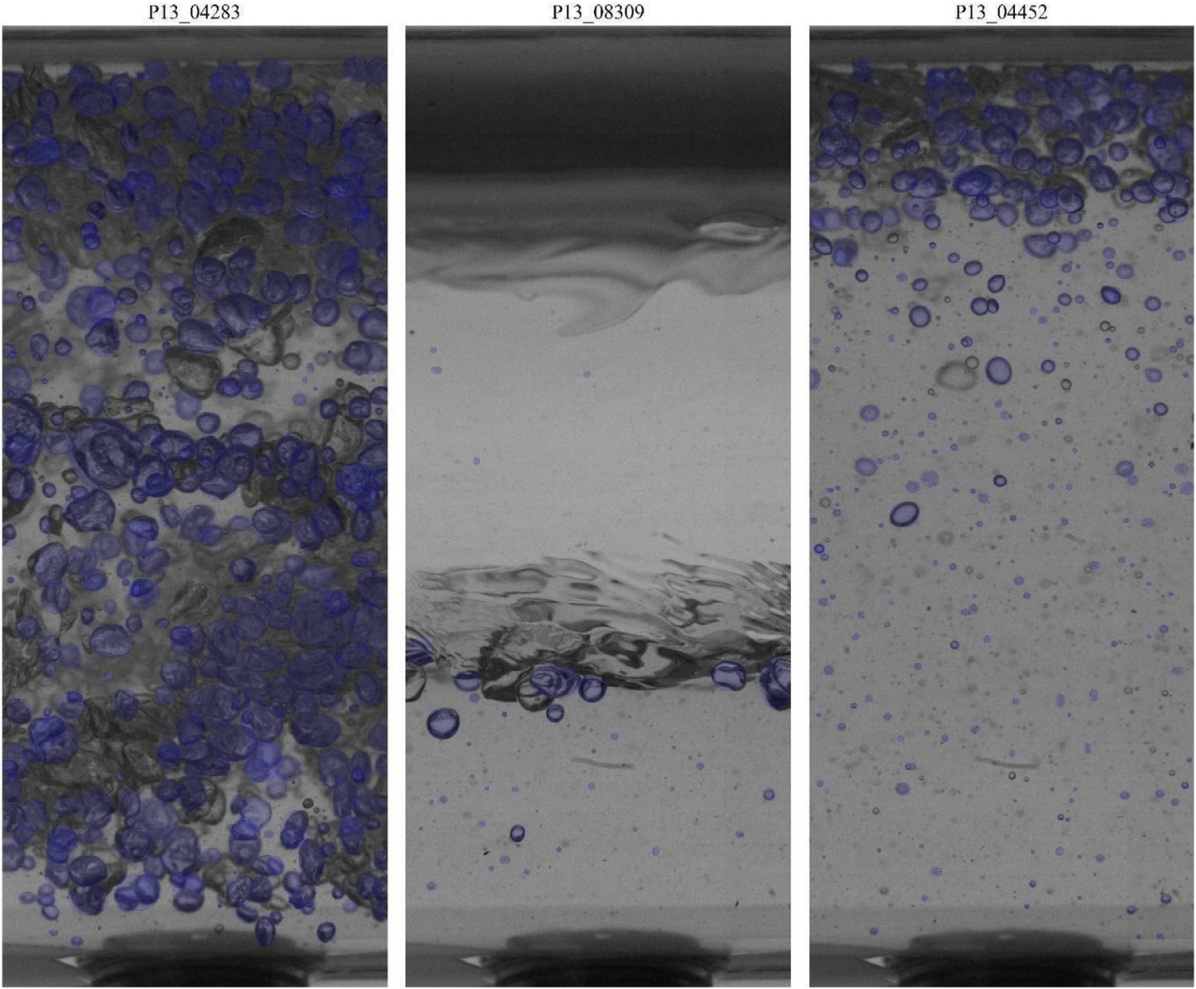
Representative images from experimental point 13, showing the bubble contours (blue lines) automatically identified by the Segment Anything Model (SAM).

The automatic processing of the high-velocity camera images was performed using the SAM to identify and segment bubbles in each frame. The output of this process is stored in the bubble_contours variable, which consists of a list of arrays, where each array contains the (x, y) coordinates of the pixels that define the perimeter of an individual bubble. Fig. 5 displays three representative images from experimental point 13 where these computed contours are visualized as overlaid blue lines, showcasing the precision of the automatic segmentation process in delineating each identified bubble.

Manual Image Segmentation is a data annotation process where a human expert, rather than an automated algorithm, delineates the boundaries of objects of interest within a digital image. This method is critically important for creating a high-fidelity ``ground truth'' dataset, which serves as a gold standard for training and validating automated segmentation models. It is also employed in complex scenarios where algorithms may fail due to factors like low contrast, irregular shapes, or overlapping objects. In this work, manual segmentation was used to generate such a ground-truth dataset for individual bubbles, following a systematic procedure. The process was based on the visual inspection of a moving temporal window of three consecutive frames (*i-1, i*, and *i* *+* *1*):

1. Identification and Confirmation: An operator would first identify a bubble in the central frame (*i*). Its presence and correspondence were then visually verified in the adjacent frames (*i-1, i*, and *i* *+* *1*) to confirm its identity and rule out potential noise or image artifacts.
2. Contour Delineation: Once confirmed, the precise contour of the bubble in frame (*i*) was manually traced, distinguishing its boundary from the surrounding liquid phase.
3. ID Assignment for Tracking: A unique identifier (ID) was assigned to each delineated bubble. This same ID was associated with the corresponding bubble in the neighboring frames, formally establishing its track over the short sequence. This procedure was repeated for multiple bubbles.

**Fig. 6:**
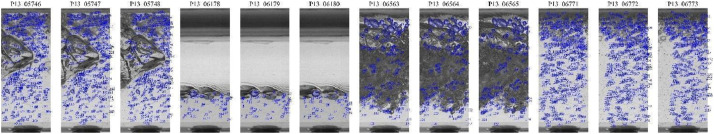
Manually segmented images from each region of the slug for experimental point 13, with the identified bubbles showing their respective identification numbers.

Manual image segmentation was performed exclusively at points within the high-passage-frequency slug flow pattern, specifically points 8–19, 21, and 22. For these points, segmentation was performed manually, with careful selection of only 12 images, chosen to capture three of each characteristic region of slug flow: the nose region, the stratified region, the mixing region, and the liquid piston region. In each region, three consecutive frames were segmented, the previous frame (*i-1*), the reference frame (*i*), and the subsequent frame (*i* *+* *1*), for example, P13_05746, P13_05747, and P13_05748, as shown in Fig. 6, where the identified bubbles are highlighted with their respective identification numbers. The manual nature of the segmentation process ensures that it is highly targeted and precise, enabling a detailed analysis of the slug flow dynamics. Each segmented image in the Dataset_800Hz has a corresponding ‘Segmentation of bubbles [-]’ data, which is structured as a dictionary containing detailed information about each identified bubble. This information, as previously described, includes a unique identifier (ID of segmented bubble), angular orientation (angle of the segmented bubble), centroid coordinates (CxCy of the segmented bubble), and the major and minor axes lengths of an ellipse fitted to the bubble (DaDb of the segmented bubble).

**Fig. 7:**
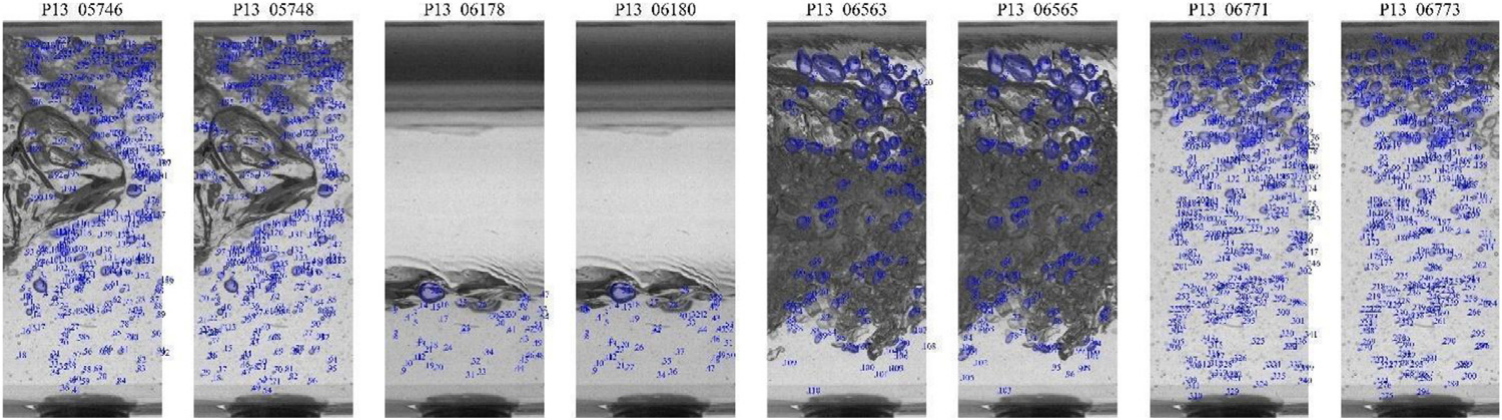
Manually tracked images from each region of the slug for experimental point 13, with the identified bubbles showing their respective identification numbers.

The tracking methodology involved comparing bubbles in two consecutive frames in the same region of the Taylor bubble flow, in which they were manually segmented. These frames included the previous frame (*i-1*) the reference frame (*i*), and the subsequent frame (*i* *+* *1*). To determine the similarity between the bubbles, the absolute error in area and velocity was calculated. Bubbles were considered potential pairs if the area error was within 50 % and the velocity error within 85 %, using the Taylor bubble velocity as a reference parameter. If both conditions were met, the bubbles were considered similar and classified as pairs, allowing for the identification and tracking of the same bubbles in consecutive frames within the different regions of the flow. As shown in Fig. 7, the tracked frames are always the previous frame (*i-1*) and the subsequent frame (*i* *+* *1*) relative to the reference frame (*i*), totaling 8 tracked frames. Each tracking image has an associated dictionary, referred to as ‘Tracking bubbles’, containing detailed information about the tracked bubbles. This information, as previously described, includes unique identifiers, centroid coordinates, ellipse-fitted axes, angular orientation, and velocity components (magnitude, vertical, and horizontal), as well as the bubble's surface area.

It is important to note that due to technical issues during the experimental procedure, the videos for experimental point 12 are not available. However, the recordings from the other sensors are in perfect condition.

## 4 Experimental Design, Materials and Methods

**Fig. 8:**
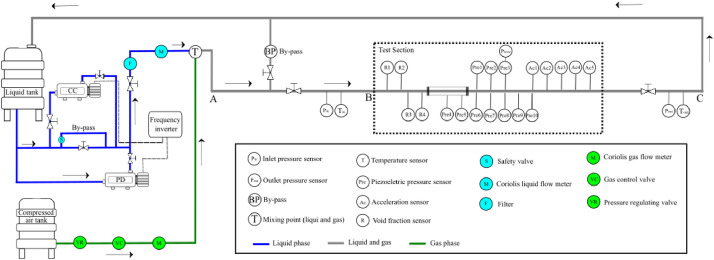
General layout of the experimental apparatus, the lines in green represent the passages of gas phase and the lines in blue represent the liquid phase and the gray lines are the passages of two-phase flow, and the test section is highlighted.

The experimental setup was built at *Laboratório Experimental de Petróleo Dr. Kelsen Valente*", in one of the labs of the Artificial Lift and Flow Assurance Research Group at the Center for Energy and Petroleum Studies (CEPETRO) of the *Universidade Estadual de Campinas* (UNICAMP). Fig. 8 depicts the schematic layout of the experimental apparatus, featuring a 24-meter flow development section preceding the measurement zone (distance between points A and B). The total pipeline length, measured from points A and C, is 36.72 m To ensure operational safety, a bypass line is incorporated to prevent the test section pressure from exceeding 4.5 bar. This safety limit is enforced by the bypass, which diverts flow and alleviates pressure buildup within the test section. The experimental setup further comprises the following components:

□ There are two different pumps for the liquid phase: a centrifugal one (CC) (Itap 65–330/2 model) for high liquid flow rates and a positive displacement (PD) (Netzsch NM090–02S model) one for low flow rates. These pumps are activated individually depending on the experimental point, and are controlled by a frequency inverter (WEG CFW – 700 model).
□ The liquid is pumped from the tank to the liquid line (blue line) by one of the two pumps, its mass flow rate is measured with a Coriolis flowmeter ($M_{L}$) (MicroMotion F300S355CCAZPZZZZ model).
□ Compressed air is supplied from the gas line (green line) at a pressure of 8 bar, a pressure regulator (Festo VPPM-6L-l-1-G18–0L10H-A4P model) and its mass flow controlled by a needle valve (HORA Regelarmaturen MC 160/230 model). The mass flow rate is measured by a Coriolis flowmeter ($M_{G}$) (MicroMotion CMFS050M313N2BGPKZZ model). Consequently, the air is introduced into the liquid line through a simple T-mixture.
□ Manometric pressure is gauged at the inlet ($P_{in}$), test section ($P_{test}$), and pipeline end ($P_{out}$), to correct compressed air properties.
□ Temperature readings ($T_{in}$ and $T_{out}$) are taken at the inlet and outlet of the test section.
□ Differential pressure measurements (DP) were acquired using sensors positioned across the test section, with a 3 m separation between the pressure tap locations. This configuration enables accurate determination of the pressure drop along this segment of the pipeline.

Table 3 summarizes the properties of the liquid (water) and gas phases, including density, viscosity, and surface tension. Notably, the gas density was calculated for each experimental point using the ideal gas law, employing mean pressure and temperature values, with the resulting values included in the metadata file for each point. Additionally, the table provides detailed geometric and material properties of the steel pipe used in the experiments, such as internal diameter, thickness, inclination, material density, elastic modulus, and roughness.

**Table 3:** Fluid properties, pipe characteristics, and experimental setup parameters.

Properties	Value
Density - liquid phase	998 kg/m³
Density - gas phase	1.33154 - 2.80750 kg/m³
Viscosity - liquid phase	0.001 Pa.s
Viscosity - gas phase	0.000018 Pa.s
Superficial tension	0.0077 N/m
Internal diameter of pipe	0.0805 m
Thickness of pipe	0.00425 m
Inclination of pipe	0 °
Modulus of elasticity of pipe	204.40 GPa
Mass density of pipe	7337.86 kg/m³
Roughness of pipe	0.000596

**Fig. 9:**
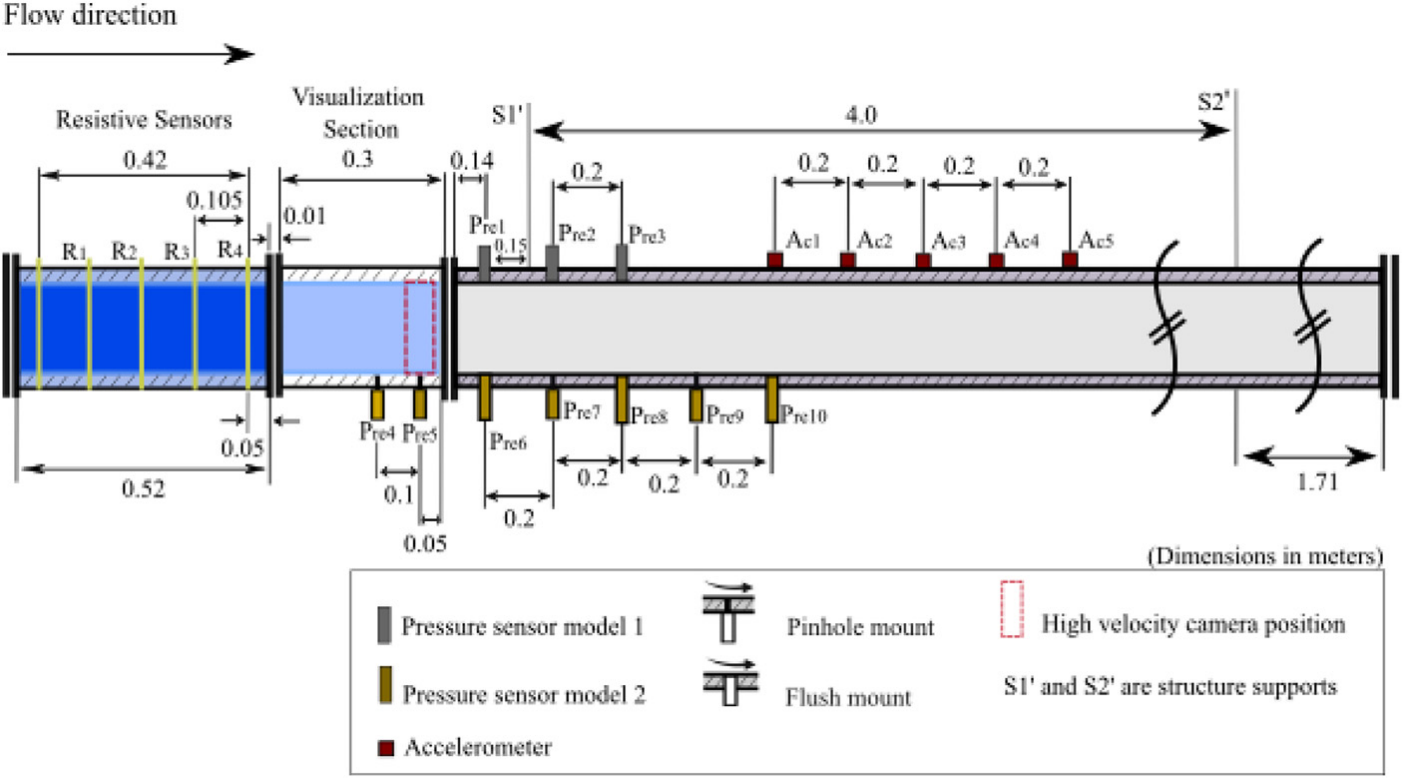
Schematic of the test section on the experimental setup. The positions and distances of the pressure and void fraction sensors are indicated as well as the visualization section where the high-velocity camera was positioned.

**Fig. 10:**
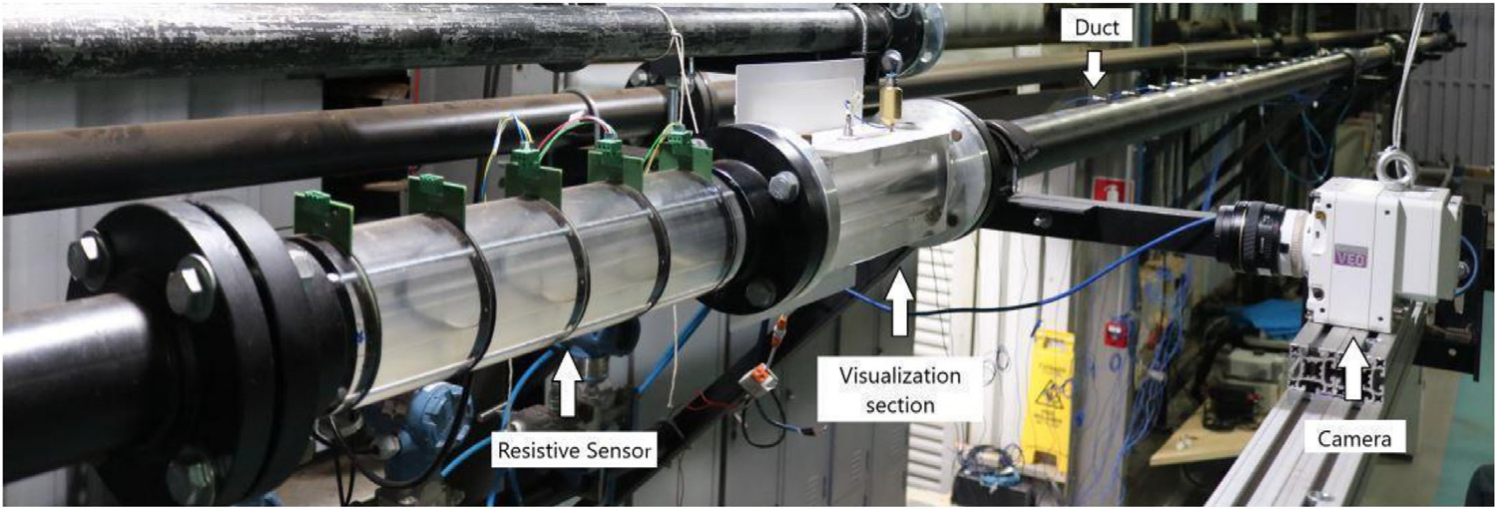
Photo of the experimental bench with the main elements highlighted [9].

Fig. 9 presents the test section that has one module of four resistive sensors ($R_{1}$, $R_{2}$, $R_{3}$, and $R_{4}$) measuring void fraction. There are two models of piezoelectric pressure sensors with two different assemblies: model 1 PCB 113B21 ($P_{re1}$, $P_{re2}$ and $P_{re3}$) and model 2 PCB 106B50 ($P_{re4}$, $P_{re5}$, $P_{re6}$, $P_{re7}$, $P_{re8}$, $P_{re9}$ and $P_{re10}$). Furthermore, pressure sensors have two different mountings: pinhole and flush. Five acceleration sensors of the same model PCB 352C33 are used ($A_{c1}$, $A_{c2}$, $A_{c3}$, $A_{c4}\;$and $A_{c5}$). Finally, a high-velocity Phantom camera model VEO 640 is positioned in front of the viewing box to capture the internal flow. Fig. 10 shows a photo of the experimental setup with the main elements highlighted.

The 106B50 and 113B21 piezoelectric pressure sensors are robust, high-sensitivity devices designed for accurate pressure measurements across a range of applications. The 106B50 model has a measurement range of 5 psi (34.45 kPa), sensitivity of 500 mV/psi (72.5 mV/kPa), and a low-frequency response of 0.5 Hz. The 113B21 model offers a measurement range of 200 psi (1379 kPa), sensitivity of 25 mV/psi (3.6 mV/kPa), and a low-frequency response of 0.5 Hz. Both models feature high resonant frequencies and fast response times, making them suitable for capturing transient pressure events. The sensors are constructed with durable materials, such as 17–4 stainless steel and a 316 L stainless steel diaphragm, ensuring long-term performance and hermetic sealing. As shown in Fig. 9, the sensors have distinct mounting configurations: one flush-mounted for direct interface with the flow and another with a pinhole mount, enabling pressure measurements in various settings. Further details on specifications, including operational temperature range, temperature coefficients, electrical characteristics, and physical dimensions, can be found in the respective model datasheets.

A conductance sensor for measuring void fraction was developed in-house employing a switched configuration among four measurement stations to prevent crosstalk, as detailed in the work of Das Neves [12]. The system operates with a sampling rate of 25.6 kHz and a switching frequency of 1600 Hz. The carrier signal is centered at a frequency of 3200 Hz, and the system produces a single output signal. This configuration ensures precise measurement and reduces interference between sensors. Although the measurement system is fast, this acquisition strategy has a direct consequence: it results in an effective acquisition frequency of 400 Hz for each individual sensor signal. According to the Nyquist Theorem, this effective rate limits the maximum frequency of the void fraction phenomenon that can be accurately analyzed to 200 Hz, explaining the limited frequency range observed in the signal's Power Spectral Density (PSD) in Fig. 2.

**Fig. 11:**
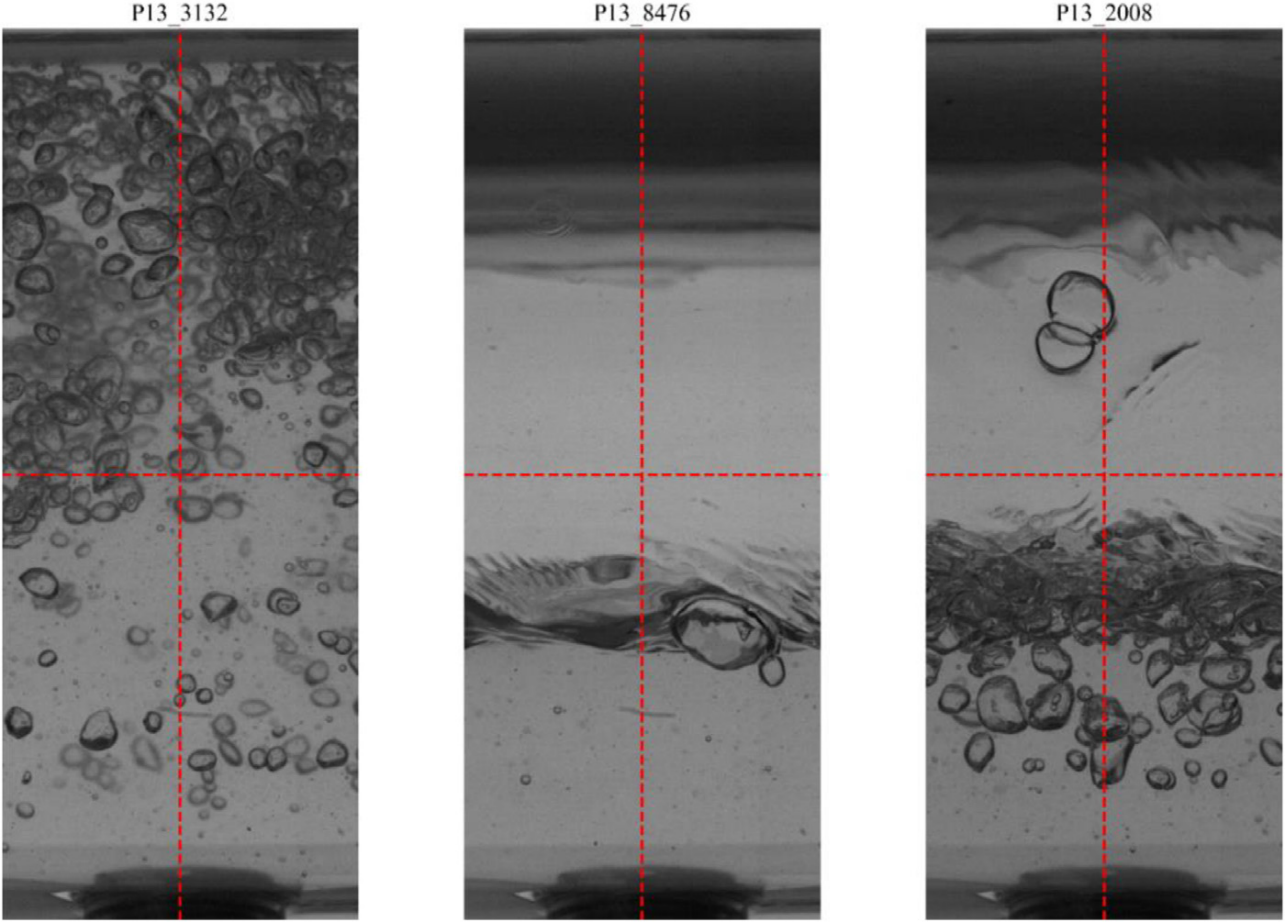
Detailed view of the test section instrumentation, including the high-velocity camera's field of view (red dashed lines) centered at 400 × 1000 pixels, which corresponds to the center of the $P_{re5}$ pressure sensor.

High-velocity images were acquired by a Phantom VEO 640 camera, operating at 800 fps and a resolution of 800 × 2000 pixels, parameters that were maintained for all experimental points. The camera was positioned upstream of the pressure sensor $P_{re5}$, as present in Fig. 9, with the field of view centered at 400 × 1000 pixels, as shown in Fig. 11. The start of the recording was commanded by a 5 V electrical pulse (trigger). Crucially, this same trigger signal was recorded along with the data from the other instruments, all sampled at the same 25.6 kHz rate, serving as a common temporal marker to ensure precise synchronization between the time series and the captured images.

Sensor signals (acceleration, pressure, and void fraction) were acquired synchronously at 25.6 kHz for 600 s. High-velocity video of the flow was recorded at 800 frames per second and synchronized with the sensor data using a trigger signal. To accommodate the disparate sampling rates and incorporate image processing results, two distinct datasets were created: one at 25.6 kHz and another at 800 Hz. Data acquisition was performed using a National Instruments (NI) system with a LabVIEW interface.

### 4.1 Flow pattern classification

**Fig. 12:**
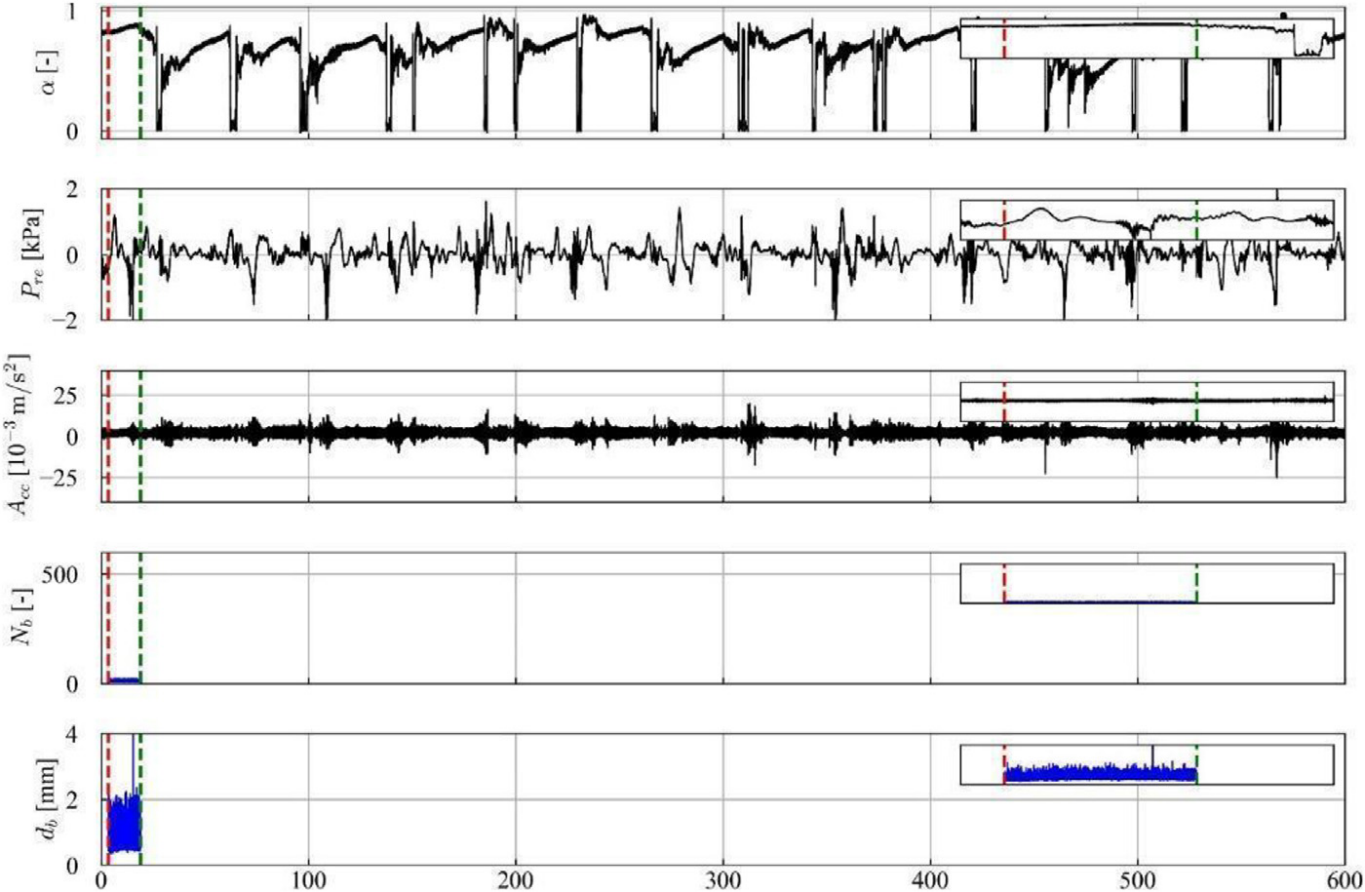
Time series data for experimental point 5, including: void fraction (α), pressure ($P_{re}$), acceleration ($A_{cc}$), number of bubbles ($N_{b}$), and bubble diameter ($d_{b}$). The red and green lines indicate the start and end of the high-velocity video recording, respectively.

**Fig. 13:**
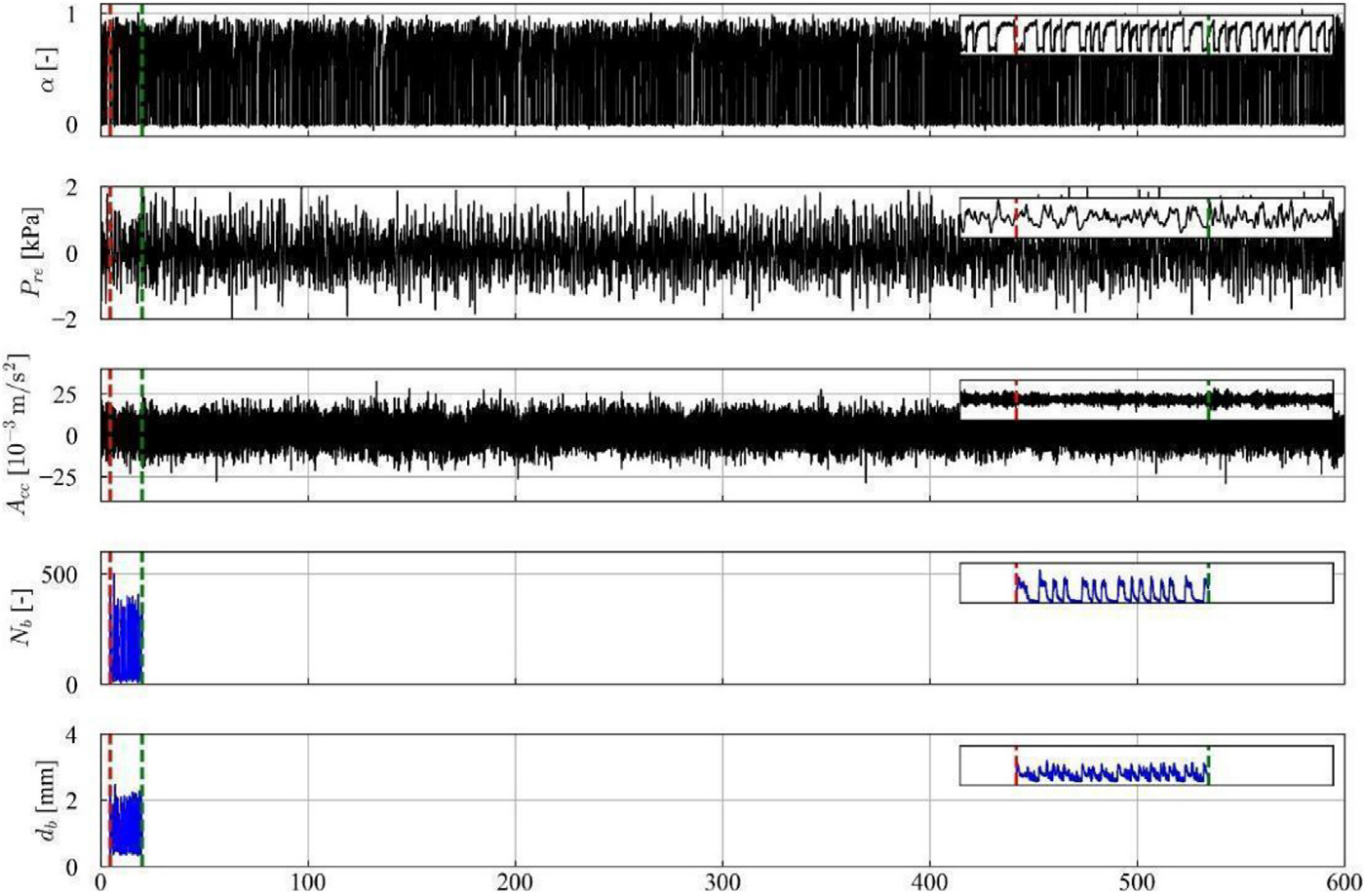
Time series data for experimental point 13, including: void fraction (α), pressure ($P_{re}$), acceleration ($A_{cc}$), number of bubbles ($N_{b}$), and bubble diameter ($d_{b}$). The red and green lines indicate the start and end of the high-velocity video recording, respectively.

Flow pattern identification was conducted using two distinct techniques: visual observation of high-velocity camera images (approximately 15 s) and analysis of void fraction sensor time series (600 s). Samples exhibiting alternating patterns of high void fraction (Taylor bubbles, values near unity) and low void fraction (liquid slugs, values near zero) were categorized as slug flow. Due to the extended duration (600 s) and low frequency of the void fraction time series resulted in the classification of most data points as slug flow, although points 1–7 exhibited a predominance of stratified regions characterized by long Taylor bubbles and short liquid slugs. Fig. 12 illustrates the void fraction, pressure and acceleration sensor time series and number and average diameter of small bubbles from image processing for experimental point 5, highlighting the low passage frequency in the void fraction series with short liquid slugs and a dominance of the stratified region. Conversely, Fig. 13 for experimental point 13 reveals a considerably higher passage frequency and a more balanced distribution of liquid slugs and Taylor bubbles.

In contrast, the limited recording time (approximately 15 s) of the high-velocity camera constrained data acquisition to the stratified flow region in points 1, 2, 3, 5, and 7, thus hindering definitive flow pattern categorization. This limitation is illustrated in Fig. 12, where the red and green vertical lines demarcate the start and end of the video recording, respectively, highlighting the exclusive capture of the stratified flow region. Points 4 and 6, exhibiting limited liquid slug occurrences, were classified as slug flow based on image analysis. Points 8–23, characterized by higher passage frequencies, provided sufficient data for flow pattern identification within the camera recording timeframe, as shown in Fig. 13.

To conclude, Table 1 consolidates the final flow pattern classifications for the 23 experimental points, resulting from a comparative analysis of two methodologies. The table presents a side-by-side summary of the results from the 600 s void fraction sensor analysis and the approximately 15 s visual inspection of the video. This presentation allows for a critical analysis of the nuances of each technique, particularly for the points marked with a '-', where a definitive pattern could not be concluded from the images because the approximately 15 s window captured only the stratified portion of a low-frequency slug flow. Therefore, Table 1 serves not only as a summary but also as a tool for understanding the intermittent nature of the flows and the complementarity between the data.

### 4.2 Void fraction determination using quick-closing valves

Void fraction measurements were conducted for each experimental point using a quick-closing valve technique, isolating a 31.4 m section of the test line between valves V1 and V2, shown in Fig. 8. Three distinct methods were employed to determine void fraction: resistive sensor measurements, high-velocity camera image analysis, and direct measurement of the drained liquid volume. The corresponding void fraction value obtained from each method is included in the metadata file for each experimental point. Table 4 summarizes the void fraction values obtained via these three methods, along with their uncertainties, demonstrating good agreement between the different techniques. These quick-closing tests were performed exclusively for points 8–19, 21, and 22, which exhibited slug flow patterns with higher passage frequencies.

**Table 4:** Comparison of void fraction measurements obtained via three different methods (resistive sensor, high-velocity camera, and fluid drainage) for experimental points 8–19, 21, and 22.

Point	$J_{sl}$ [m/s]	$J_{sg}$ [m/s]	Void fraction with rapid closure [-] (fluid drainage)	Void fraction with rapid closure [-] (image)	Void fraction with rapid closure [-] (void fraction sensor)
8	0586	0519	0,36 ± 0,02	0,35 ± 0,02	0,33 ± 0,02
9	0584	0987	0,55 ± 0,01	0,55 ± 0,03	0,59 ± 0,02
10	0592	1481	0,57 ± 0,01	0,57 ± 0,02	0,58 ± 0,02
11	1191	0382	0,19 ± 0,02	0,13 ± 0,02	0,15 ± 0,02
12	1172	0904	0250 ± 0,02	0,29 ± 0,02	0,30 ± 0,02
13	1172	0904	0,38 ± 0,02	0,39 ± 0,03	0,43 ± 0,02
14	1315	0478	0,22 ± 0,02	0,14 ± 0,02	0,17 ± 0,02
15	1266	0962	0,36 ± 0,02	0,27 ± 0,02	0,30 ± 0,02
16	1226	1661	0,37 ± 0,02	0,34 ± 0,02	0,35 ± 0,02
17	2154	0512	0,16 ± 0,02	0,08 ± 0,02	0,13 ± 0,02
18	2038	1041	0,24 ± 0,02	0,19 ± 0,02	0,23 ± 0,02
19	2005	1207	0,22 ± 0,02	0,22 ± 0,02	0,25 ± 0,02
21	2,96,673	0403	0,07 ± 0,02	0,01 ± 0,01	0,05 ± 0,02
22	2,83,054	0904	0,13 ± 0,02	0,10 ± 0,02	0,12 ± 0,02

### 4.3 Image processing

**Fig. 14:**
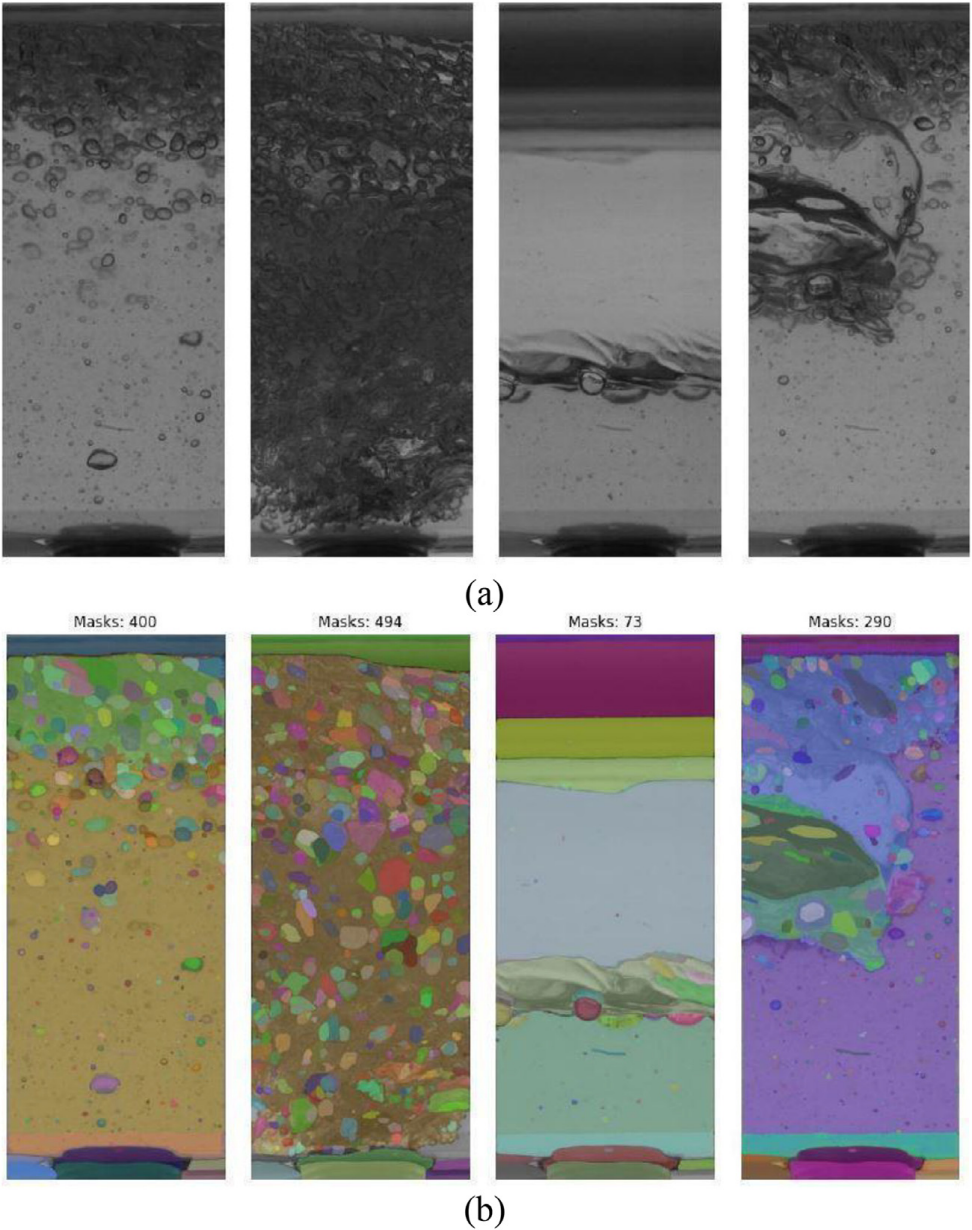
Segmentation of bubbles employing SAM is conducted for four distinct regions in the piston-like pattern at experimental point 13 of the test matrix. Displayed are the original image (a), extracted from high-velocity camera videos, and the SAM-generated mask results (b) [1].

Bubble detection in high-velocity camera images was performed using the Segment Anything Model (SAM), a promptable segmentation model capable of generating masks without requiring task-specific training or manual annotation. The employed SAM implementation utilizes a ViT-H architecture pre-trained via masked autoencoding, comprising 636 million parameters [8,1]. The segmentation process involves predicting multiple masks for sampled points within the image, followed by filtering based on quality criteria. This approach enables SAM to adapt to varying image complexities, generating different numbers of masks as needed. Fig. 14 illustrates the original images (a) and the corresponding automatically generated masks (b) for four characteristic regions of slug flow, with distinct colors representing individual masks.

**Fig. 15:**
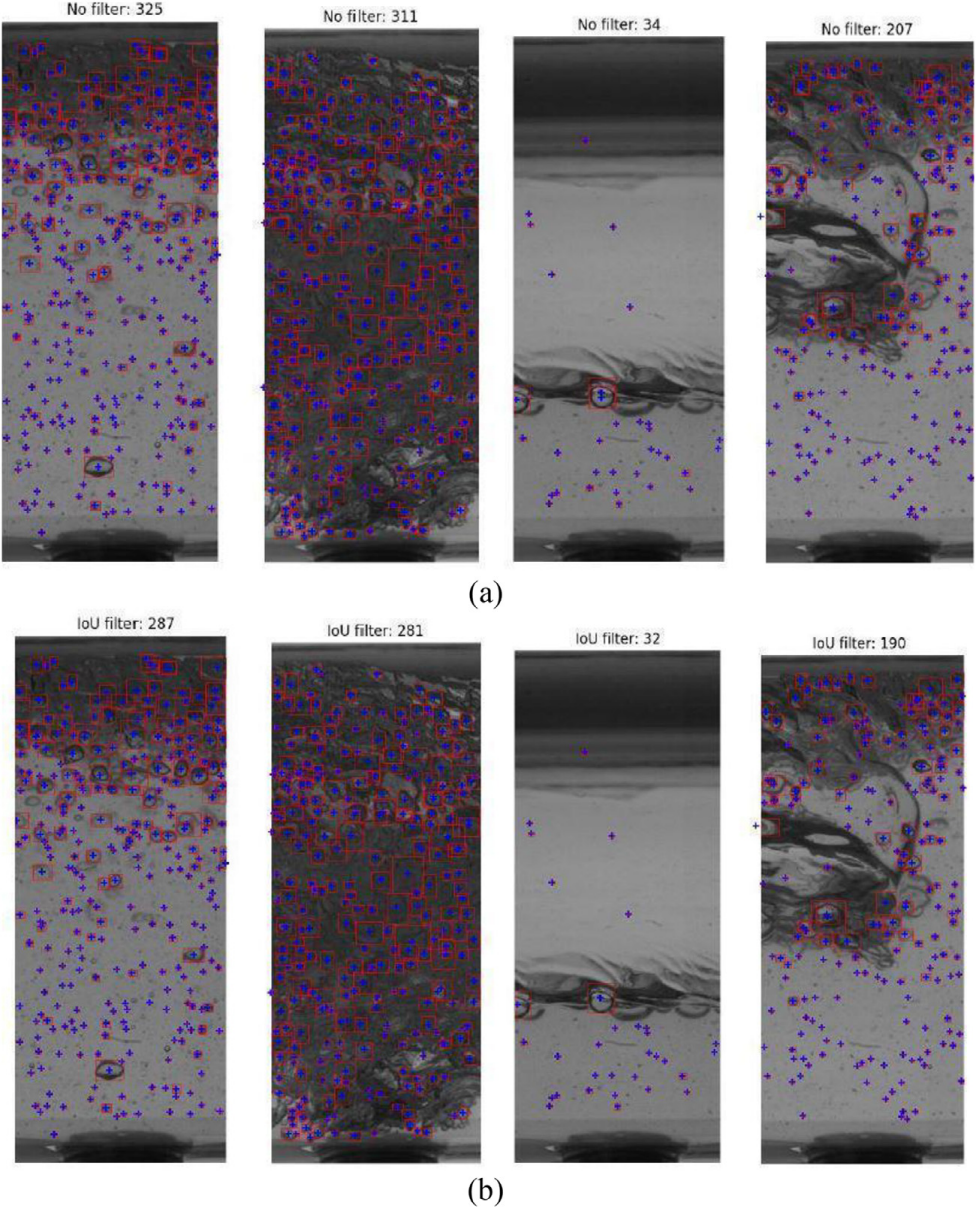
Segmentation of bubbles is performed using SAM for four distinctive regions within the Taylor bubble pattern: Taylor bubble nose, stratified region, mixing region, and liquid slug, taken from experimental point 13 of the test matrix. The outcomes of bubble detection illustrate each bubble (a), identified by red squares, and the subsequent bubble filtering using IoU (b).

The subsequent step involves extracting bubble information from identified contours. Ellipses are fitted to contours to calculate equivalent bubble diameters, and a bounding box is created for precise position and size determination. A post-processing step, employing Intersection Over Union (IoU), is used to address overlapping bounding boxes, ensuring accurate bubble detection. Fig. 15(a) presents images of each characteristic region with the identified bubbles delineated by contour boxes. It is important to note that only contours with an area compatible with the bubble size and circularity between 0.7 and 1.2 are considered. Circularity, measured by the formula $\frac{4\pi \;x\;area}{perimeter²}$, indicates how close an object is to a true circle, with 1 being the ideal circularity. Fig. 15(b) illustrates the outcome of the bubble detection process after the removal of bounding boxes and bubbles with an IoU value greater than 0.5. The adjustment process focused on retaining only those bubbles with the largest area, resulting in a reduction in the total number of bubbles.

## Limitations

It is crucial to point out that due to technical recording issues during the experimental procedure, there are no videos available for experimental point 12.

## Ethics Statements

The authors duly adhered to ELSEVIER ‘Ethics in publishing’ policy. No ethical issues are associated with this work.

## Credit Author Statement

**DA das Neves:** Writing - Original draft preparation, Investigation, Software, Validation Data Curation. **SC Vieira:** Conceptualisation, Writing - Review & Editing. **JR Cenzi:** Data Curation. AT Fabro: Conceptualisation, Methodology, Writing - Review & Editing, Supervision. **MS Castro:** Writing - Review & Editing, Resources, Supervision, Project administration, Funding acquisition.

## Data Availability

Unicamp Research Data RepositoryDataset of two-phase flow in a horizontal pipe: synchronized measurements of acceleration, pressure, void fraction and high-speed camera - Raw Data (Original data)
